# [Corrigendum] Engineered AXL^-ECD^-Fc variants that abolish the AXL/Gas6 interaction suppress tumor cell migration

**DOI:** 10.3892/ol.2026.15698

**Published:** 2026-06-11

**Authors:** Yanting Duan, Bo Hu, Chunxia Qiao, Longlong Luo, Xinying Li, Jing Wang, Hao Liu, Tingting Zhou, Beifen Shen, Ming Lv, Jiannan Feng

Oncol Lett 17: 5784–5792, 2019; DOI: 10.3892/ol.2019.10255

Following the publication of the above paper, it was drawn to the Editor's attention by an interested reader that, regarding the cell migration assay experiments shown in Fig. 3D on p. 5789, the ‘blank’ and the ‘Gas6+AXL-M2’ data panels were apparently matching, suggesting that the data in this figure had been assembled incorrectly.

Upon consulting their original data, the authors have realized that this figure was inadvertently assembled incorrectly; specifically, the images for the SKOV3 Transwell assays (for the “blank” and “Gas6+AXL-M2” experiments) were inadvertently misplaced during typesetting. The revised version of Fig. 3, now showing the correct data panel for the “Gas6+AXL-M2” experiment, is shown on the next page. The authors regret the error that was made during the compilation of the original figure, and are grateful to the editor of *Oncology Letters* for allowing them the opportunity to publish this Corrigendum. Note that this error did not have a significant impact on the conclusions reported in this study. All the authors agree with the publication of this corrigendum; furthermore, they apologize to the readership for any inconvenience caused.

## Figures and Tables

**Figure 3.**
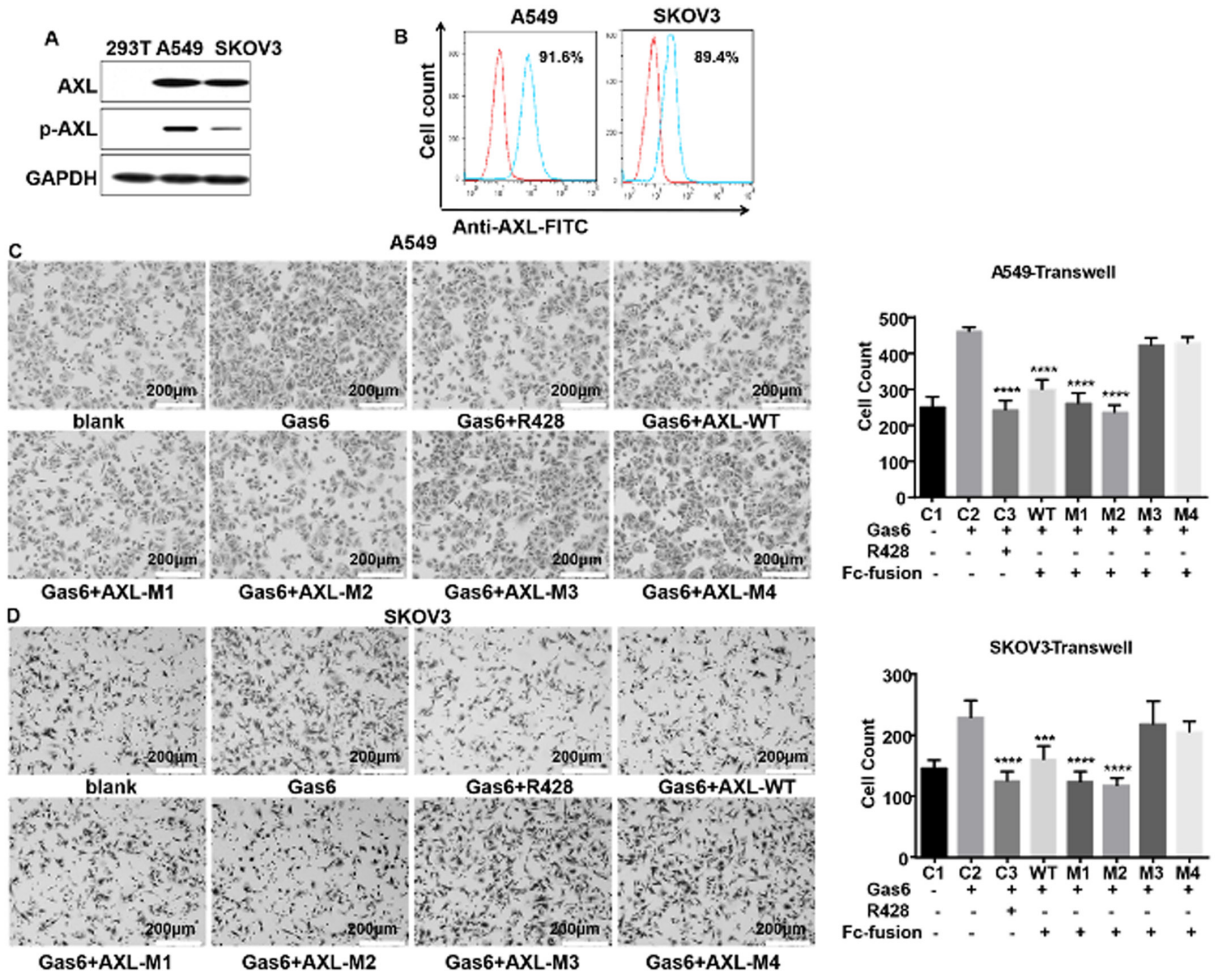
AXL−ECD-Fc-WT/M fusion proteins inhibited migration in SKOV3 and A549 cells. (A) Total AXL expression in cancer cell lines A549 (carcinoma), SKOV3 (human ovarian cancer cell) and 293T cells. (B) Flow cytometry analysis of the expression of AXL on the SKOV3 and A549 cell surface. Transwell assay results for high-affinity AXL−ECD-Fc-M1/M2 fusion proteins or low-affinity AXL−ECD-Fc-M3/M4 fusion proteins, which were added into cell culture medium, to capture free Gas6 in (C) A549 and (D) SKOV3 cells. ***P<0.005 and ****P<0.0001 vs. Gas6 (C2 group). AXL, AXL receptor tyrosine kinase ligand; Gas6, growth arrest-specific 6.

